# Exploiting Compositionally Similar Grape Marc Samples to Achieve Gradients of Condensed Tannin and Fatty Acids for Modulating In Vitro Methanogenesis

**DOI:** 10.3390/molecules23071793

**Published:** 2018-07-20

**Authors:** Josh L. Hixson, Zoey Durmic, Joy Vadhanabhuti, Philip E. Vercoe, Paul A. Smith, Eric N. Wilkes

**Affiliations:** 1 The Australian Wine Research Institute, P.O. Box 197, Glen Osmond SA 5064, Australia; paul.smith@wineaustralia.com (P.A.S.); eric.wilkes@awri.com.au (E.N.W.); 2 School of Agriculture and Environment, The University of Western Australia M085, 35 Stirling Hwy, Crawley WA 6009, Australia; zoey.durmic@uwa.edu.au (Z.D.); joy.vadhanabhuti@uwa.edu.au (J.V.); philip.vercoe@uwa.edu.au (P.E.V.); 3 Institute of Agriculture, The University of Western Australia M085, 35 Stirling Hwy, Crawley WA 6009, Australia

**Keywords:** condensed tannin, bioactivity, methanogenesis, grape marc, fatty acids, in vitro batch fermentation

## Abstract

Ruminants produce large amounts of the greenhouse gas, methane, which can be reduced by supplementing feed with products that contain anti-methanogenic compounds, such as the solid winemaking by-product, grape marc. The aim of this study was to exploit compositional differences in grape marc to better understand the roles of condensed tannin and fatty acids in altering methanogenesis in a ruminant system. Grape marc samples varying in tannin extractability, tannin size and subunit composition, and fatty acid or tannin concentrations were selected and incubated in rumen fluid using an in vitro batch fermentation approach with a concentrate-based control. Four distinct experiments were designed to investigate the effects on overall fermentation and methane production. Generally, fatty acid concentration in grape marc was associated with decreased total gas volumes and volatile fatty acid concentration, whereas increased condensed tannin concentration tended to decrease methane percentage. Smaller, extractable tannin was more effective at reducing methane production, without decreasing overall gas production. In conclusion, fatty acids and tannin concentration, and tannin structure in grape marc play a significant role in the anti-methanogenic effect of this by-product when studied in vitro. These results should be considered when developing strategies to reduce methane in ruminants by feeding grape marc.

## 1. Introduction

Methane is inherently produced in ruminant livestock systems, but there are options for limiting the emissions of this greenhouse gas (GHG). Changes to feeding systems and the addition of ruminant dietary supplements can alter digestion away from high methane production and towards capturing more energy for the animal with lower GHG emissions [1,2,3,4]. With an increasing global need for food, and finite resources on which to draw, agricultural by-products are a valuable input into feeding systems [5], especially when these can also have positive effects on GHG emissions. Diet supplementation with feed containing condensed tannins (CTs) has shown promise in reducing methane emissions from livestock, and the link between tannin-containing feed and reductions in methane intensity have been noted previously [6]. The CTs are a diverse class of compounds, and more recent attention has turned away from the concentration-dependent model of CT dosing and focused more closely on the structure, composition, and extractability of CT to better understand the observed responses, be it for methanogenesis [7,8], protein binding capacity [9,10], or exploiting anthelmintic properties [11].

Grape marc, a solid material remaining after wine making, contains significant proportions of CT [12], and has been the topic of some interest in supplementing a ruminant diet [13,14,15,16,17,18,19]. The CT found in this winemaking by-product has been extensively surveyed and varies in both concentration and subunit composition [12]. While the concentration is dependent on the natural variability in the wine grape from which it is derived, the processing that grape marc is exposed to is also key in determining the concentration and extractability of CT that remains. For example, red grape marc that is present during alcoholic fermentation undergoes a higher level of extraction than white grape marc, and subsequent thermal treatments at processing plants can further decrease CT concentrations. Grape marc contains very small amounts of extractable tannin, if any, and mainly contains tannin that is loosely bound to cell walls [20]. Many studies linking CT to methanogenesis have exploited either CT-containing extracts, or a whole plant material that contains CT [6,7,8]. Very little work has focused on by-product feeding where the soluble CT has been largely removed, and the CT under investigation is bound in some way to cell wall material. In addition to variable CT concentration and composition, a considerable proportion of grape marc dry matter (DM) contains seed-derived fatty acids [12], which have also been widely implicated in reducing methanogenesis [13,21,22]. In grape marc this component is commonly referred to as “crude fat” or simply “fat” [13,23]; however, it will herein be referred to as fatty acid (FA) concentration to avoid confusion.

Winemaking and subsequent processing steps result in grape marc with a highly varied composition [12]. As such, establishing the role that CT or FA from grape marc plays in methanogenesis over another component may be difficult to determine by simply comparing individual grape marc samples. An alternative experimental approach is to study single compounds through the addition or removal of those components and generate samples that are varied in the desired species. However, many compounds of interest in grape marc are not readily extractable and are well incorporated into cell wall fibers. As such, additive studies can produce systems that fail to mimic naturally occurring interaction with cell wall material. Conversely, extractive removal of a suspected bioactive can affect numerous compounds. For grape marc, this methodology may not accurately replicate these species in a natural state.

This study aimed to identify grape marc samples that differ specifically in CT or FA concentration, or CT composition, but are otherwise compositionally similar. After identifying these samples, the aim was to produce blends or comparisons that result in changes of the single, desired variable, allowing for simplified investigations of key grape marc components with respect to methanogenesis. This approach was used to determine how the extractability, composition, or concentration of tannin, as well as the concentration of FA, affects the overall microbial fermentability and production of methane in an in vitro batch fermentation system.

## 2. Results and Discussion

### 2.1. Selection and Profile of Grape Marc Samples

Five grape marc samples (GM1, GM6, GM14, GM18, GM20) and a commercial enological tannin (GT) were selected for in vitro fermentation experiments based on their CT and FA concentrations, and CT compositional profiles (Table 1). To study the impact of CT extractability on methanogenesis grape marc 6 (GM6), a post-steam distillation mixture of red and white grape marc, was selected. This sample displayed moderate amounts of both CT and FA, and contained no water extractable tannin (WET). The addition of commercially available extractable tannin (GT) to this marc provided a comparison of native, loosely-bound tannin with exogenous, extractable tannin on methane abatement (Experiment 1).

**Figure 1:**
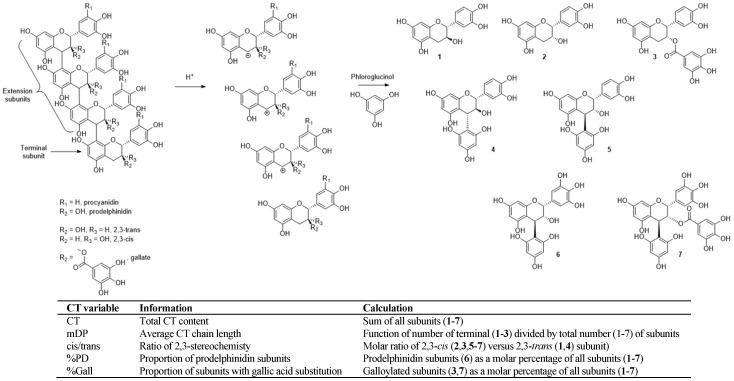
Acid-catalyzed depolymerization of a condensed tannin chain followed by reaction with phloroglucinol to yield identifiable subunits that are found in grape-derived CT, and subunits involved in calculating CT variables from phloroglucinolysis.

Much like the observed changes in CT extractability due to processing, the origin of CT is similarly important for determining CT composition. Skins and seeds from fresh grapes both provide structurally distinct CT [24,25], which is also the case for grape marc-derived CT [12]. Skin-derived CT is generally larger and consists of more subunits (higher mean degree of polymerization, mDP), is higher in both subunit cis/trans ratio (cis/trans) and percentage of prodelphinidin subunits (%PD), and lower in the extent of gallic acid substitution (%Gall). Conversely, seed-derived CT is compositionally opposed for these factors (lower mDP, %PD and cis/trans, and higher %Gall) (Figure 1). However, simply blending skin and seed samples does not isolate CT composition as a sole variable, as the desired CT compositional changes occur alongside FA concentration changes that inherently exist for skin- versus seed-derived samples [12].

To overcome this, GM20, derived from grape stalk, was selected as it showed a unique composition with low FA concentrations (like marc skin), but also a seed-like CT composition. Therefore, blending a marc skin-derived sample, GM18, with GM20 provided a gradient in CT composition, specifically mDP, cis/trans ratio, and %Gall, without substantial changes in total CT concentration or FA (Experiment 2).

GM20 also provided an appropriate comparison against marc seed (GM14), as the relatively similar CT concentration and composition allowed for comparison of high- and low-FA grape marc samples, and for examination of differing FA concentrations on methane production (Experiment 3). Conversely, the impact of tannin and/or FA on methanogenesis was investigated by keeping the FA concentrations similar and varying the CT concentration. The extent of processing that created GM1 resulted in a very low CT concentration, without significantly impacting on FA content. A blend from GM14 to GM1 created a gradient of CT concentration, while FA concentration was held relatively constant (Experiment 4).

These selected grape marc samples and blends were incubated in sheep rumen fluid using an in vitro batch fermentation approach and compared against a concentrate-based control substrate. More detailed information of each treatment and resulting composition can be found in the Supplementary Material (Table S1).

### 2.2. Experiment 1—Extractability of CT and Methanogenesis

Inclusion of GM6 had no effect on the total gas production and volatile fatty acid (VFA) concentration (Table 2), although there was a significant reduction in methane volume (*p* < 0.05) compared with the control. Inactivating the CT by addition of polyethylene glycol (GM6 + PEG) gave a slight recovery in gas volume, and the methane volume increased back to that of the control, suggesting that the slight reductions observed could be attributed to the presence of CT. The addition of extractable tannin (GM6 + GT) provided slight reductions in methane percentage (mL/100 mL of total gas) compared with GM6, without having a significant effect on overall fermentation. The addition of PEG (GM6 + GT + PEG) only recovered the gas volume to that observed for GM6, and not back to that of the control, bringing into question the impact of PEG on completely inhibiting CT in this treatment. The addition of extractable tannin provided the lowest methane production (volume and percentage) without affecting the total gas volume, although these changes were not statistically different from that observed with loosely bound CT present in GM6.

The investigation into the role of CT extractability in methanogenesis also inherently explored tannin composition. The extractability of grape-derived tannin changes with tannin size, as larger CT interacts more strongly with cell wall material, removing it from solution [26]. As a result, the distribution of tannin remaining in solution becomes skewed towards smaller CT. Due to the correlation between grape marc CT variables, modulation in tannin size (mDP) inherently favors CT of a lower cis/trans ratio and %PD, and higher %Gall. As such, any changes in methanogenesis observed in Experiment 1 may relate to compositional changes (i.e., smaller CT) in addition to the level of extractability.

Experiment 1 showed inconsistencies in the inhibition of CT by PEG, especially when both extractable and cell wall-bound tannin were present. Initially, it was concluded that the extractable CT present bound with all the PEG, leaving none available to interact with the loosely bound CT portion. In this study, PEG additions were made at 350 mg per ferment, which in the case of GM6 + GT + PEG (with a total of 34.9 mg of CT) represented a ratio of 10:1 (*w*/*w* PEG:CT). This was equivalent to the ratio used in previous work with extractable tannin [7], but much less than in experiments exploiting native CT in whole plant material (between 100:1 and 1000:1) [8,27]. In one study it was also noted that the fermentation response derived from CT inactivation by PEG was not as expected [8]. As such, the role of PEG in ferments containing both extractable tannin and cell wall-bound tannin is unclear, and warrants further investigation. In this study, however, the behavior of PEG may have been a response to the low rate of PEG addition, and higher concentrations may be required for cell wall-bound CT than extractable CT.

### 2.3. Experiment 2—Composition of CT and Methanogenesis

The inclusion of GM18 into the control did not produce a significant reduction in gas production, VFA concentration, or methane variables. However, as the inclusion was moved across a gradient from GM18 to GM20, significant fermentation changes were observed, most notably for methane volume with GM18 + GM20 at 1:2 ratio (Table 3). While reductions in total gas volume across this gradient also occurred, the same effect was not seen for VFA concentration. Moving from treatment GM18 to GM20, a trend for reductions in methane percentage was seen with decreasing mDP and cis/trans ratio. Treatments that contained higher proportions of GM20 also possessed higher concentrations of WET (Table 1). This experiment likely mirrors Experiment 1 in exploiting CT extractability as well as composition, a relationship that cannot be uncoupled.

Here, minimal variation in the %PD was created across the grape marc gradient (20–24.7%PD), a factor that has previously been linked with CT-derived reductions in methanogenesis [7,8]. Reported in vitro batch fermentation assessment of grape marc samples have ranged from 3.9 to 33.0%PD [23], and %PD has been observed as high as 48.4% for CT from skin-only marc [12]. Comparatively, when %PD has been linked to methanogenesis, the CT assessed ranged from 3.3 to 99.2%PD in one study [7], and 52.7 to 94.8%PD in the second [8]. As such, even if isolation of %PD in grape marc CT as a sole variable was achievable, the range available may not provide a high enough %PD required to generate comparative reductions in methane.

### 2.4. Experiment 3 and 4—CT and/or FA Concentration and Methanogenesis

Separating the effect of tannin and FA on methanogenesis was achieved in two separate experiments, one comparing high-CT samples with differing FA content (Experiment 3), and another blending high-FA samples across a gradient of increasing CT concentration (Experiment 4).

In Experiment 3, a 30% inclusion of GM20 yielded similar gas production and VFA concentration to the control, but gave a significant reduction in both methane volume and percentage (Table 4). The addition of PEG (GM20 + PEG) provided no recovery in methane production, suggesting either that tannin is not as anti-methanogenic as previously thought, or as noted in Experiment 1, that the mechanism of action of PEG with grape marc CT is not as simple as previously suggested. The inclusion of GM14 (high FA sample) resulted in a significant reduction in the gas volume and VFA concentration from the control, which is consistent with earlier results that high-FA grape marc retards fermentation, specifically gas production [23]. The addition of PEG (GM14 + PEG) had no significant effect on gas volume and VFA concentration, while methane percentage was closer to that of the control. These results suggest that CT are more heavily impacting methane production, and the reductions observed in total gas production are due to the presence of FA. The comparison between GM20 (high CT, low FA) and GM14 (high CT, high FA) showed a similar trend, as methane percentage was not significantly affected by the increase in FA, unlike overall gas volume and methane volume.

In Experiment 4, as well as the gradient in CT, the blend of GM1 and GM14 in this experiment also provided an increase in ME and a slight increase in FA content (see Table 1). All grape marc inclusions gave significant reductions in gas volume and VFA production from the control (Table 5), which aligned with previous results in this study and others using grape marc containing high levels of fatty acids [23]. There was no obvious increase in gas production or VFA concentration with increasing ME across the gradient. However, the significant drop in methane percentage observed from the control to GM1 provided evidence for the anti-methanogenic property of FA. In the case of ruminant digestion, reductions in methane gas production may be beneficial as this represents an energy loss to the system that could be directed into animal performance, rather than lost as gaseous emissions [4]. However, here the fermentations that contained high FA grape marc samples (GM1 and GM14) also resulted in lower VFA concentrations, suggesting that it may not be a redirection of energy, but rather just a reduction in the extent of fermentation and a net loss of energy to the system. Shifting the inclusion towards GM14 and higher CT concentrations yielded significant reductions in methane volume and percentage, while gas production and VFA concentration remained relatively steady. This again, highlighted the anti-methanogenic properties of CT without yielding a negative effect on the extent of fermentation.

### 2.5. The Grape Marc Blending Methodology

The use of compositionally similar grape marc samples to understand how individual components alter in vitro methanogenesis in these experiments has provided some clarity despite the complexity of grape marc composition. In our previous compositional survey of grape marc [12], principal component analysis of the analytical outcomes produced a model containing 42 variables that were significant in contributing to the difference between those 20 samples, many of which were co-correlated. Table 6 outlines the relationship between key compositional parameters of those 20 samples that were of consequence here, either the bioactive compounds of interest (CT and FA), or other metrics that were expected to heavily influence fermentation parameters, such as fiber composition, fermentable sugars, and metabolizable energy.

It has already been stated that grape marc CT compositional variables are well correlated, and further investigation of compositional correlations show that many other parameters are also significantly correlated. For example, the size of CT (mDP) correlates with most other parameters with a high level of statistical significance. This creates an inability to select multiple samples that display differences in a single variable as there exists an inherent difference in many other key fermentation-relevant variables. It appears the processing differences that create compositional change in grape marc, in addition to the natural variation that is observed in CT, makes grape marc an unsuitable candidate for this simplified methodology.

## 3. Materials and Methods

### 3.1. Grape Marc and Grape-Derived Tannin

Five different grape marc samples were selected from a pool of 20 diverse grape marc samples that had been thoroughly characterized [12]. Samples were chosen based on their tannin and fatty acid content; these were a steam-distilled and flash-dried marc (GM1), a steam-distilled mix of red and white marc (GM6), a seed-only sample from fresh white marc (GM14), a skin-only sample from fresh red marc (GM18), and a grape stalk-only sample (GM20). Grape marc collection, storage, and preparation were as previously described. Grape marc sample identifiers were consistent with previous work on these same samples [12,23]. Extractable grape-derived tannin, Grap’tan PC (GT), was obtained commercially (EnolTech, Angaston, South Australia, Australia) and used as supplied.

### 3.2. Chemical Analysis

All grape marc samples were analyzed for tannin chemistry, nutritive value, and a range of other important factors as previously described [12], with all sample identifiers matching those used in previous publications. Only key analytical outcomes (tannin, fatty acids, energy) have been re-published here. Extractable grape-derived CT (Grap’tan PC) was analyzed for CT subunit concentration and composition by phloroglucinolysis as previously described [24]. The composition of GM blends submitted to fermentation was calculated from the individual composition, and either the mass ratio of substrates used for concentration-based components (CT, FA, ME etc.), or using the mass ratio of CT for CT compositional variables (mDP, cis/trans, %PD and %Gall). The full composition of each treatment used in this work can be seen in Supplementary Material (Table S1), along with the fatty acid profiles (Table S2).

### 3.3. In Vitro Fermentation

The use of donor animals was approved by the Animal Ethics Committee of The University of Western Australia, Approval number RA3/100/1424. The in vitro batch fermentation experiments were run as previously described, with some modifications [28]. In brief, the control fermentation substrate was a commercial pellet (Milne Standard Pellets, Milne Feed, Welshpool, Western Australia, Australia) that contained barley (350 g/kg), oats (200 g/kg), wheat (200 g/kg), lupins (60 g/kg), straw (100 g/kg), mill mix (50 g/kg), and minerals (40 g/kg); and had nutritive value of dry matter (DM) 910 g/kg DM, acid detergent fiber 156 g/kg DM, neutral detergent fiber 282 g/kg DM, starch 310 g/kg DM, crude protein 145 g/kg DM, and crude fat 12 g/kg DM. Pellet material was ground to pass a 1 mm screen prior to inclusion in the assay.

Each treatment was tested in triplicate. Fermentation substrate (control, 0.5 g) was weighed into specialized anaerobic culturing vials (100 mL serum bottle, Cat. No. W012465I Wheaton, Millville, NJ 08332, USA) and transferred into an anaerobic chamber (Coy Vinyl Anaerobic Chamber; Coy Laboratory Products Inc., Grace Lake, MI, USA, maintained at 39 °C and supplied with 800 mL/L N_2_, 100 mL/L CO_2_ and 100 mL/L H_2_). The rumen fluid was collected on the day of the experiment 3 h after feeding to obtain a sample with a maximal microbial activity from three ruminally cannulated adult Merino wethers (mean body weight 65.4 ± 2.0 kg) that were fed a diet consisting of 1 kg oaten chaff, 250 g lupins, and 25 g mineral mix for 2 weeks before sampling. Rumen fluid was pooled, strained, buffered using McDougall buffer (1:1.5 *v*/*v*), and the pH adjusted to between 7.1 and 7.3 using citric acid. Each tube was filled with 50 mL of this buffered rumen fluid. For grape marc treatments, 0.35 g of a control substrate was mixed with 0.15 g of grape marc or grape marc blend. Where extractable tannin was used, 10 mg was added, and polyethylene glycol-containing treatments were supplemented with 350 mg of PEG 8000. Tubes were stoppered, crimped, and incubated with shaking for 24 h. At the end of the incubation period, gas and methane volume and methane concentrations in the headspace gas expressed as mL/g dry matter incubated (DMi), as well as concentrations of volatile fatty acids (VFA), ammonia, and acetate:propionate ratios were measured as described previously [28]. Gas production was measured using a pressure transducer (Greisinger Electronic GmbH, Regenstauf, Germany), and methane production was determined by gas chromatography with Thermal Conductivity Detector (Bruker-450 GC, Bruker Technologies, Melbourne, Australia) equipped with Compass CDS Acquisition software (Bruker Technologies, Australia), using a Hayesep Q 0.25 mm × 10 m column with He as carrier gas flow at 30 mL/min. Data shown are an average of triplicate ferments, results from individual replicates can be found in Supplementary Material (Table S3).

### 3.4. Statistical Analyses

Values shown in the text are the average of triplicates. Analysis of variance (*p* = 0.05) was performed using Tukey’s multiple comparison test (GraphPad Prism, GraphPad Software, La Jolla, CA, USA). Pearson correlation coefficients and corresponding *p*-values were determined from data taken from Hixson et al. (2016) [12] using an X-Y correlation analysis (GraphPad Prism).

## 4. Conclusions

The methodology used here provided a preliminary insight into the compounds present in grape marc that are responsible for modulating methanogenesis. We have investigated the role that grape marc CT and FA play in methanogenesis. In general, treatments containing high FA concentrations resulted in significant reductions in gas production and VFA concentration, and any reductions in methane volume were largely attributed to this drop in overall gas production. When considering CT, a trend for smaller, more extractable tannin being more effective at reducing methanogenesis was observed.

## Figures and Tables

**Table 1:** Key compositional information for selected grape marc samples (upper panel), as determined by Hixson et al. (2016) [12], and relative compositional change between grape marc samples being compared in this work (lower panel).

Sample	Type	CT (g/kg DM)	Tannin Composition				WET (g/kg DM)	FA (g/kg DM)	CP (%DM)	ADF (%DM)	NDF (%DM)	ME (MJ/kg)	Comments
			mDP	cis/trans	%PD	%Gall							
GM1	Steam distilled, dried	20.05	N/A				0	115.9	12.1	56.1	61.4	6.61	Low CT, high FA
GM6	Red/white spent	78.36	10.00	10.16	14.3%	9.1%	0	67.1	12.7	34.9	42.6	10.25	Typical processed marc
GM14	White seed	126.13	6.87	8.27	6.1%	16.1%	47.32	152.1	11.5	41.1	51.3	9.41	High CT, high FA
GM18	Red skin	120.74	32.55	26.00	24.7%	3.9%	0	14.0	8.3	16.4	19.4	11.72	High CT (large), low FA
GM20	Red stalk	114.79	9.98	9.22	20.0%	6.1%	40.65	5.2	3.2	26.3	34.1	9.20	High CT (small), low FA
Grap’tan PC (GT)	Grape-derived tannin extract	574.39	5.92	5.17	5.5%	16.0%							
**Relative composition change between samples for comparison**													
GM20/GM18	Experiment 2	95.1	30.7	35.5	80.9	155.9	N/A	37.3	38.6	160.4	175.8	78.6	
GM14/GM20	Experiment 3	109.9	68.8	89.6	30.3	265.0	116.4	2907.5	359.4	156.3	150.4	102.3	
GM14/GM1	Experiment 4	629.1	N/A	N/A	N/A	N/A	N/A	131.2	95.0	73.3	83.6	142.4	

Numbers shown in red indicate desired compositional change to be achieved in the experiment. CT (condensed tannin concentration, as determined by phloroglucinoysis); mDP, mean degree of polymerization; %PD, percentage of prodelphinidin-type subunits; %Gall, percentage of subunits with gallic acid substitution; WET, water extractable tannin; CP, crude protein; ADF, acid detergent fiber; NDF, neutral detergent fiber; ME, metabolizable energy; DM, dry matter.

**Table 2:** In vitro fermentation parameters for Experiment 1; grape marc samples of differing tannin extractability.

Treatment	Description	Gas Volume (mL/g DM)	CH_4_ Volume (mL/g DM)	CH_4_% (mL/100 mL Total Gas)	VFA (mmol/L)	Ac:Pr	NH_3_ (mg/L)
Control	Control	290.8 ± 6.1	39.60 ± 1.31 ^a^	13.62 ± 0.17 ^a^	90.46 ± 2.33 ^a^	2.997 ± 0.006 ^a^	160.4 ± 6.2 ^a^
GM6	Loosely bound CT	264.4 ± 19.4	33.03 ± 2.54 ^b^	12.45 ± 0.08 ^a,b^	84.02 ± 0.61 ^a,b^	3.123 ± 0.012 ^b,c^	142.8 ± 0.0 ^c^
GM6 + PEG	Removal of CT	288.8 ± 3.4	38.83 ± 1.65 ^a^	13.46 ± 0.64 ^a^	84.31 ± 2.12 ^a,b^	3.057 ± 0.025 ^a,b^	152.8 ± 1.8 ^a,b^
GM6 + GT	Addition of extractable CT	267.4 ± 0.8	32.43 ± 1.00 ^a^	11.89 ± 0.34 ^b^	81.44 ± 1.26 ^b^	3.153 ± 0.060 ^c^	114.8 ± 2.5 ^d^
GM6 + GT + PEG	Removal of CT	269.6 ± 8.9	34.73 ± 2.84 ^a,b^	12.58 ± 0.66 ^a,b^	80.42 ± 4.29 ^b^	3.107 ± 0.025 ^b,c^	150.0 ± 1.2 ^b,c^
SEM		13.78	2.738	0.609	3.374	0.0436	4.29
*p*		0.0217	0.0018	0.0037	0.0045	0.001	<0.0001

Data expressed as mean value ± standard deviation of triplicates. Values in the same column within each experiment with different superscript letters were significantly different (*p* < 0.05). GM, grape marc; PEG, polyethylene glycol; GT, Grap’tan extractable tannin; CT (condensed tannin concentration, as determined by phloroglucinoysis); VFA, volatile fatty acid; Ac:Pr, molar ratio of acetate to propionate; DM, dry matter.

**Table 3:** In vitro fermentation parameters for Experiment 2; gradient of grape marc samples with differing tannin composition.

Treatment	Description	Gas Volume (mL/g DM)	CH_4_ Volume (mL/g DM)	CH_4_% (mL/100 mL Total Gas)	VFA (mmol/L)	Ac:Pr	NH_3_ (mg/L)
Control	Control	290.8 ± 6.1 ^a,b^	39.60 ± 1.31 ^a^	13.62 ± 0.17 ^a^	90.46 ± 2.33	2.997 ± 0.006 ^a^	160.4 ± 6.2 ^a^
GM18	High mDP and cis/trans CT	295.9 ± 3.5 ^a^	38.23 ± 1.16 ^a^	12.91 ± 0.28 ^a,b^	84.49 ± 6.82	2.780 ± 0.036 ^b^	112.0 ± 3.9 ^b^
GM18 + GM20 (2:1)	Medium-high mDP and cis/trans CT	296.7 ± 2.1 ^a^	37.87 ± 0.29 ^a,b^	12.75 ± 0.03 ^a,b^	85.17 ± 2.51	2.800 ± 0.026 ^b^	105.2 ± 3.7 ^b,c^
GM18 + GM20 (1:2)	Medium-low mDP and cis/trans CT	275.9 ± 11.3 ^b^	33.57 ± 3.14 ^b^	12.06 ± 0.63 ^b^	82.97 ± 2.04	2.840 ± 0.070 ^b^	96.0 ± 3.2 ^c^
GM20	Low mDP and cis/trans CT	288.3 ± 2.5 ^a,b^	35.37 ± 1.10 ^a,b^	12.23 ± 0.42 ^b^	85.56 ± 5.74	2.823 ± 0.021 ^b^	95.6 ± 9.7 ^c^
SEM		8.39	2.310	0.505	5.99	0.0526	8.00
*p*		0.0125	0.008	0.0031	0.3502	0.0003	<0.0001

Data expressed as mean value ± standard deviation of triplicates. Values in the same column within each experiment with different superscript letters were significantly different (*p* < 0.05). GM, grape marc; mDP, mean degree of polymerization; CT (condensed tannin concentration, as determined by phloroglucinoysis); VFA, volatile fatty acid; Ac:Pr, molar ratio of acetate to propionate; DM, dry matter.

**Table 4:** In vitro fermentation parameters for Experiment 3; grape marc samples of differing fatty acid concentrations.

Treatment	Description	Gas Volume (mL/g DM)	CH_4_ Volume (mL/g DM)	CH_4_% (mL/100 mL Total Gas)	VFA (mmol/L)	Ac:Pr	NH_3_ (mg/L)
Control	Control	290.8 ± 6.1 ^a^	39.60 ± 1.31 ^a^	13.62 ± 0.17 ^a^	90.46 ± 2.33 ^a^	2.997 ± 0.006 ^a^	160.4 ± 6.2 ^a,b^
GM20	High CT, low FA	288.3 ± 2.5 ^a^	35.37 ± 1.10 ^b^	12.23 ± 0.42 ^b,c^	85.56 ± 5.74 ^a,b^	2.823 ± 0.021 ^b^	95.6 ± 9.7 ^c^
GM20 + PEG	Removal of CT, FA effect only	295.2 ± 2.1 ^a^	36.47 ± 0.75 ^b^	12.35 ± 0.17 ^b,c^	87.63 ± 0.63 ^a^	2.767 ± 0.015 ^b^	105.6 ± 3.6 ^c^
GM14	High CT, high FA	266.5 ± 2.4 ^b^	31.40 ± 0.76 ^c^	11.76 ± 0.18 ^b^	79.40 ± 2.11 ^b^	3.030 ± 0.046 ^a^	145.2 ± 3.6 ^a^
GM14 + PEG	Removal of CT, FA effect only	271.5 ± 3.5 ^b^	34.47 ± 0.55 ^b^	12.67 ± 0.20 ^c^	78.03 ± 1.06 ^b^	2.963 ± 0.055 ^a^	174.8 ± 6.2 ^b^
SEM		4.99	1.280	0.339	4.084	0.0468	8.58
*p*		<0.0001	<0.0001	<0.0001	0.0019	<0.0001	<0.0001

Data expressed as mean value ± standard deviation of triplicates. Values in the same column within each experiment with different superscript letters were significantly different (*p* < 0.05). GM, grape marc; PEG, polyethylene glycol; FA, fatty acids; CT (condensed tannin concentration, as determined by phloroglucinoysis); VFA, volatile fatty acid; Ac:Pr, molar ratio of acetate to propionate; DM, dry matter.

**Table 5:** In vitro fermentation parameters for Experiment 4; gradient of grape marc samples with differing condensed tannin concentration.

Treatment	Description	Gas Volume (mL/g DM)	CH_4_ Volume (mL/g DM)	CH_4_% (mL/100 mL Total gas)	VFA (mmol/L)	Ac:Pr	NH_3_ (mg/L)
Control	Control	290.8 ± 6.1 ^a^	39.60 ± 1.31 ^a^	13.62 ± 0.17 ^a^	90.46 ± 2.33 ^a^	2.997 ± 0.006 ^a^	160.4 ± 6.2 ^a^
GM1	Low CT, high FA	268.1 ± 4.6 ^b^	34.23 ± 0.95 ^b^	12.72 ± 0.23 ^b^	78.03 ± 3.26 ^b^	3.077 ± 0.046 ^a,b^	156.8 ± 6.6 ^a,b^
GM1 + GM14 (2:1)	Medium-low CT, high FA	269.3 ± 7.1 ^b^	32.97 ± 0.25 ^b,c^	12.21 ± 0.23 ^c,d^	76.68 ± 4.04 ^b^	3.113 ± 0.012 ^b^	152.0 ± 3.7 ^a,b^
GM1 + GM14 (1:2)	Medium-high CT, high FA	270.1 ± 0.9 ^b^	33.30 ± 0.26 ^b,c^	12.30 ± 0.06 ^b,c^	78.51 ± 0.66 ^b^	3.050 ± 0.026 ^a,b^	146.8 ± 3.5 ^b^
GM14	High CT, high FA	266.5 ± 2.4 ^b^	31.40 ± 0.76 ^c^	11.76 ± 0.18 ^d^	79.40 ± 2.11 ^b^	3.030 ± 0.046 ^a,b^	145.2 ± 3.6 ^b^
SEM		6.59	1.115	0.250	3.741	0.0438	6.72
*p*		0.0006	<0.0001	<0.0001	0.0006	0.011	0.0157

Data expressed as mean value ± standard deviation of triplicates. Values in the same column within each experiment with different superscript letters were significantly different (*p* < 0.05). GM, grape marc; CT (condensed tannin concentration, as determined by phloroglucinoysis); VFA, volatile fatty acid; Ac:Pr, molar ratio of acetate to propionate; DM, dry matter.

**Table 6:** Pearson correlation coefficients (r) for grape marc compositional variables, with *p*-value shown in parentheses, using data for twenty grape marc samples taken from Hixson et al., 2016 and re-assessed to give correlations within the sample set.

Variable	CT	mDP	cis/trans	%PD	%Gall	FA	ADF	NDF	NFC	ESC	ME
**mDP**	0.14 (0.5624)										
**cis/trans**	−0.04 (0.886)	0.94 (<0.0001)									
**%PD**	0.17 (0.476)	0.70 (0.0009)	0.59 (0.0084)								
**%Gall**	−0.03 (0.9157)	−0.70 (0.0009)	−0.69 (0.0011)	−0.71 (0.0006)							
**FA**	0.00 (0.9915)	−0.58 (0.0086)	−0.58 (0.0091)	−0.62 (0.0045)	0.93 (<0.0001)						
**ADF**	0.05 (0.8262)	−0.73 (0.0004)	−0.72 (0.0005)	−0.64 (0.0035)	0.81(<0.0001)	0.82 (<0.0001)					
**NDF**	0.04 (0.8744)	−0.74 (0.0003)	−0.74 (0.0003)	−0.67 (0.0017)	0.88(<0.0001)	0.87 (<0.0001)	0.97 (<0.0001)				
**NFC**	0.10 (0.6837)	0.73 (0.0004)	0.70 (0.0008)	0.70 (0.0008)	−0.87 (<0.0001)	−0.87 (<0.0001)	−0.96 (<0.0001)	−0.98 (<0.0001)			
**ESC**	0.14 (0.5633)	0.66 (0.0022)	0.65 (0.0027)	0.55 (0.014)	−0.74 (0.0003)	−0.70 (0.0008)	−0.90 (<0.0001)	−0.90 (<0.0001)	0.93 (<0.0001)		
**ME**	−0.26 (0.273)	0.52 (0.0212)	0.57 (0.0117)	0.37 (0.1189)	−0.70 (0.0009)	−0.66 (0.0021)	−0.77 (0.0001)	−0.80 (<0.0001)	0.68 (0.0014)	0.62 (0.0048)	
**Lignin**	0.09 (0.7108)	−0.60 (0.0062)	−0.59 (0.0072)	−0.61 (0.0052)	0.86 (<0.0001)	0.93(<0.0001)	0.94 (<0.0001)	0.93 (<0.0001)	−0.92 (<0.0001)	−0.78 (0.0001)	−0.74 (0.0003)

CT (condensed tannin concentration, as determined by phloroglucinoysis), mDP, mean degree of polymerization; %PD, percentage of prodelphinidin-type subunits; %Gall, percentage of subunits with gallic acid substitution; FA, fatty acid; ADF, acid detergent fiber; NDF, neutral detergent fiber; NFC, non-fiber carbohydrate; ESC, ethanol soluble carbohydrate; ME, metabolizable energy; DM, dry matter.

## Supplementary Materials

The following are available online. Table S1. The composition of each grape marc treatment used in this work. Table S2. The fatty acid profiles of the grape marc samples used in this work, as determined in Hixson et al. 2016 [1]. Table S3. Raw in vitro batch fermentation outputs for each replicate.
