# Supplementary material for: Exploiting Compositionally Similar Grape Marc Samples to Achieve Gradients of Condensed Tannin and Fatty Acids for Modulating In Vitro Methanogenesis
Source: Molecules. 2018 Jul 20;23(7):1793. doi: 10.3390/molecules23071793 (PMC6100490; doi:10.3390/molecules23071793)
Supplement: Supplementary file 1 [file molecules-23-01793-s001.pdf]

Table S1. The composition of each grape marc treatment used in this work.

Sample	Type	CT (g/kg DM)	Tannin composition mDP	cis/trans	%PD	%Gall	WET (g/kg DM)	FA (g/kg DM)	CP (%DM)	ADF (%DM)	NDF (%DM)	ME (MJ/kg)
Control	Concentrate-based pellet	0.0	N/A				0.0	12.0	14.5	15.6	28.2	11.0
GM6	Experiment 1	23.5	10.0	10.2	14.3%	9.1%	0.0	28.5	14.0	21.4	32.5	10.8
GM6 + GT		23.5	8.7	8.5	11.5%	11.3%	11.4	28.5	14.0	21.4	32.5	10.8
GM18	Experiment 2	36.2	32.5	26.0	24.7%	3.9%	0.0	12.6	12.6	15.8	25.6	11.2
GM18 + GM20 (2:1)		35.6	25.0	20.4	23.1%	4.6%	4.1	11.7	12.1	16.8	27.0	11.0
GM18 + GM20 (1:2)		35.0	17.5	14.8	21.6%	5.4%	8.1	10.8	11.6	17.8	28.5	10.7
GM20		34.4	10.0	9.2	20.0%	6.1%	12.2	10.0	11.1	18.8	30.0	10.5
GM20	Experiment 3	34.4	10.0	9.2	20.0%	6.1%	12.2	10.0	11.1	18.8	30.0	10.5
GM14		37.8	6.9	8.3	6.1%	16.1%	14.2	54.0	13.6	23.3	35.1	10.5
GM1	Experiment 4	6.0	N/A				0.0	43.2	13.8	27.8	38.2	9.7
GM1 + GM14 (2:1)		16.6	N/A				4.7	46.8	13.7	26.3	37.2	10.0
GM1 + GM14 (1:2)		27.2	N/A				9.5	50.4	13.7	24.8	36.1	10.2
GM14		37.8	N/A				14.2	54.0	13.6	23.3	35.1	10.5

CT (condensed tannin concentration, as determined by phloroglucinoysis), mDP, mean degree of polymerisation; %PD, percentage of prodelphinidin-type subunits; %Gall, percentage of subunits with gallic acid substitution; WET, water extractable tannin; FA, fatty acid; CP, crude protein; ADF, acid detergent fibre; NDF, neutral detergent fibre; ME, metabolizable energy; DM, dry matter.

Table S2. The fatty acid profiles of the grape marc samples used in this work, as determined in Hixson et al. 2016 [1].

Sample	Content, percentage of total fatty acid								Degree of unsaturation	Degree of polyunsaturation
	Palmitic C16	Palmitoleic C16:1	Stearic C18	Oleic C18:1 cis 9	Linoleic C18:2 n6	Linolenic C18:3 n3	Behenic C22:0	Lignoceric C24:0		
GM1	8.3	0.2	3.6	14.0	72.1	1.2	0.4	0.3	87.4	73.2
GM6	11.0	0.0	4.0	15.2	65.8	2.3	1.0	0.7	83.3	68.1
GM14	6.3	0.0	3.1	15.5	74.6	0.3	0.1	0.0	90.5	74.9
GM18	23.4	0.0	4.7	12.9	40.8	8.6	5.4	4.2	62.3	49.4
GM20	21.1	0.0	4.5	10.8	35.4	13.5	11.3	3.5	59.6	48.8

1. Hixson, J. L.; Jacobs, J. L.; Wilkes, E. N.; Smith, P. A., Survey of the Variation in Grape Marc Condensed Tannin Composition and Concentration and Analysis of Key Compositional Factors. J Agric Food Chem 2016, 64, (38), 7076-86, 10.1021/acs.jafc.6b03126.

Table S3. Raw *in vitro* batch fermentation outputs for each replicate.

Treatment	Replicate	Gas vol (mL/g DM)	CH_4 (mL/g DM)	CH_4 (ml/100 mL total gas)	VFA (mmol-L)	A:P	NH_3 (mg-L)
Control	1	284.01	38.142	13.43	88.182	2.9976	153.6
Control	2	295.89	40.508	13.69	92.841	2.9863	162.0
Control	3	292.44	40.210	13.75	90.345	3.0026	165.6
GM6	1	241.99	30.054	12.37	84.118	3.1279	142.8
GM6	2	275.85	34.371	12.46	83.359	3.1140	142.8
GM6	3	275.44	34.582	12.53	84.571	3.1319	142.8
GM6 + PEG	1	288.29	37.247	12.92	83.651	3.0588	154.8
GM6 + PEG	2	292.33	38.773	13.29	82.594	3.0339	151.2
GM6 + PEG	3	285.67	40.450	14.16	86.682	3.0793	152.4
GM6 + GT	1	266.79	31.321	11.51	81.717	3.1642	114.0
GM6 + GT	2	268.28	33.193	12.13	82.542	3.0886	112.8
GM6 + GT	3	267.06	32.797	12.04	80.057	3.2117	117.6
GM6 + GT + PEG	1	278.16	36.801	12.92	75.643	3.0769	150.0
GM6 + GT + PEG	2	260.48	31.528	11.82	83.921	3.1286	148.8
GM6 + GT + PEG	3	270.05	35.879	13.00	81.701	3.1104	151.2
GM18	1	292.54	36.934	12.60	91.172	2.7372	109.2
GM18	2	295.76	38.803	13.12	84.756	2.8062	110.4
GM18	3	299.49	38.993	13.02	77.534	2.7860	116.4
GM18 + GM20 (2:1)	1	294.48	37.650	12.76	84.705	2.8128	102.0
GM18 + GM20 (2:1)	2	298.75	38.227	12.77	87.871	2.7706	104.4
GM18 + GM20 (2:1)	3	296.72	37.713	12.71	82.917	2.8188	109.2
GM18 + GM20 (1:2)	1	280.85	34.814	12.30	83.226	2.7742	99.6
GM18 + GM20 (1:2)	2	283.93	35.892	12.54	80.811	2.9080	93.6
GM18 + GM20 (1:2)	3	263.05	29.976	11.35	84.860	2.8450	94.8
GM20	1	286.89	36.537	12.71	91.254	2.8379	97.2
GM20	2	291.14	35.281	12.07	85.657	2.8043	85.2
GM20	3	286.89	34.266	11.92	79.766	2.8294	104.4
GM20 + PEG	1	296.17	36.488	12.32	88.350	2.7840	105.6
GM20 + PEG	2	292.71	35.682	12.19	87.358	2.7684	102.0
GM20 + PEG	3	296.58	37.162	12.53	87.183	2.7470	109.2
GM14	1	263.86	30.589	11.57	77.862	2.9801	145.2
GM14	2	268.68	32.091	11.92	81.808	3.0397	148.8
GM14	3	267.01	31.454	11.78	78.537	3.0668	141.6
GM14 + PEG	1	273.79	34.223	12.45	79.115	2.8959	180.0
GM14 + PEG	2	267.44	34.113	12.73	77.008	2.9869	168.0
GM14 + PEG	3	273.23	35.082	12.84	77.952	2.9973	176.4
GM1	1	263.86	33.867	12.81	81.420	3.1327	157.2
GM1	2	272.96	35.255	12.89	74.917	3.0536	150.0
GM1	3	267.60	33.476	12.46	77.736	3.0454	163.2
GM1 + GM14 (2:1)	1	276.68	33.174	11.99	76.437	3.1006	151.2
GM1 + GM14 (2:1)	2	268.84	32.956	12.21	80.826	3.1214	156.0
GM1 + GM14 (2:1)	3	262.48	32.717	12.44	72.768	3.1171	148.8
GM1 + GM14 (1:2)	1	270.96	33.525	12.37	78.919	3.0709	142.8
GM1 + GM14 (1:2)	2	269.23	33.047	12.25	78.852	3.0573	148.8
GM1 + GM14 (1:2)	3	270.09	33.366	12.28	77.753	3.0168	148.8

VFA, volatile fatty acids; Ac:Pr, molar ratio of acetate to propionate.
